# Using the Lessons Learned From the Clinic to Improve the Preclinical Development of Antibody Drug Conjugates

**DOI:** 10.1007/s11095-014-1536-7

**Published:** 2014-10-23

**Authors:** Dowdy Jackson, David Stover

**Affiliations:** Agensys, Inc, 1800 Stewart Street, Santa Monica, California 90404 USA

**Keywords:** antibody drug conjugate, preclinical, site specific

## Abstract

The treatment options for cancer patients include surgery, chemotherapeutics, radiation therapy, antibody therapy and various combinations of these therapies. The challenge with each therapy is finding the balance between maximizing the anti-tumor efficacy while minimizing the dose limiting toxicities. Antibodies, unlike small molecule chemotherapeutics, selectively bind to cell surface tumor antigens and can be used to deliver radionucleotides or small molecule chemotherapeutic drugs directly to the tumor. Advances in antibody engineering, linker chemistry and the identification of potent cytotoxic drugs led to the recent approval of two antibody drug conjugates to treat breast cancer and lymphoma patients. We will discuss how the observations from the clinical development of antibody drug conjugates can guide the preclinical development of the next generation of antibody drug conjugates.

## Introduction

Small molecule chemotherapeutics and antibodies are commonly used to treat cancer patients. Small molecule chemotherapeutics, such as doxorubicin, paclitaxel and vinblastine, are delivered systemically, have relatively short serum half-lives and do not specifically target tumor cells. These molecules not only kill rapidly dividing tumor cells but they can also kill some normal cells, which results in various dose limiting toxicities (DLTs), such as neutropenia. DLTs reduce the amount of drug that can be administered to a patient which can reduce the effectiveness of a drug.

Antibodies are also delivered systemically but tend to have long serum half-lives and selectively bind to tumor cells. Antibody drug conjugates (ADCs) are a combination of the small molecule chemotherapeutic and the antibody thus allowing the selective delivery of the small molecule chemotherapeutic drugs to tumor cells and not normal cells.

Although average Drug/Antibody Ratios (DAR) are typically in the range from 2 to 4 drugs per antibody, most ADCs in clinical development today are a mix of antibodies carrying 0 to as many as 12 cytotoxic drugs covalently conjugated to specific amino acids on the antibody *via* a chemical linker. ADCs bind to cell surface antigens; they are typically internalized by the cells and processed by the lysosomes where the cytotoxic drugs are released into the cytotsol. Once the drugs are released from the lysosomes, they bind to their intracellular target, disrupt a critical cellular process and kill the cell.

The preclinical development of ADCs involves several steps which include the identification of the tumor antigen, the discovery and characterization of an antibody against the tumor antigen, the identification of the appropriate cytotoxic drug, the conjugation of the cytotoxic drug to the antibody and the characterization of the amount of aggregate and other physiochemical properties of the ADC. The preclinical evaluation of ADCs includes antibody/antigen binding studies, *in vitro* cytotoxic studies, *in vivo* anti-tumor efficacy studies, pharmacokinetic and the toxicology studies in rodent and non-human primates.

The observations from the clinical development of ADCs have been crucial in refining the preclinical development of ADCs. Improvements in antibody engineering, potency of cytotoxic drugs and improvements in the linker chemistry lead to the current generation of ADCs. We will discuss how data from the current clinical studies can be used to improve the preclinical development of the next generation of ADCs.

## ADCs: A Historical Perspective

Paul Ehrlich, the German physician and scientist, described the concept of delivering a toxophore, a cytotoxic drug, selectively to tumors. ADCs are the embodiment of this concept. The first generation of ADCs used common chemotherapeutic drugs such as methotrexate, vinblastine and doxorubicin as cytotoxic drug payloads. KS1/4 and BR96 were the first antibodies to enter clinical development as ADCs.

KS1/4 was a murine IgG2a antibody against a 40 and 42 kD glycoprotein expressed by the human lung adenocarcinoma cell line, UCLA-P3 (1). The KS1/4 antigen is expressed by several cancers including ovarian, lung, pancreatic and colorectal cancers. KS1/4 was conjugated to methotrexate (KS1/4-methotrexate) or vinblastine (KS1/4-DAVLB) (2,3). There were 6 molecules of methotrexate and 4 to 6 molecules of vinblastine per antibody on lysines using hemisuccinate linkers. Preclinical *in vivo* anti-tumor efficacy was reported for the KS1/4-methotrexate and the KS1/4-DAVLB ADCs but no significant clinical responses were observed. Patients treated with the KS1/4 antibody or KS1/4 ADCs produced an antibody response against the mouse antibody, also known as a human anti mouse antibody (HAMA) response. Although the HAMA response has been reported to result in rapid systemic clearance of the antibody thus rendering the antibody or in this case ADC ineffective, high serum levels of the KS1/4 antibody were reported in patients treated with the higher doses of the KS1/4 antibody or ADCs. Subsequent ADCs used chimeric, humanized or fully human antibodies to reduce the patient’s immune response against the antibody.

BR96-Doxorubicin (SGN-15) was licensed by Seattle Genetics from Bristol-Meyer Squibb (BMS) (4). SGN-15 was a chimeric antibody against the Lewis Y (CD174) antigen that was conjugated to doxorubicin (adriamycin) using an acid labile, 6-maleimidocaproyl hydrazone linker (5,6). In preclinical studies, SGN-15 was able to selectively kill Lewis Y expressing cells in both *in-vitro* cytotoxicity and in *in-vivo* tumor efficacy studies yet it was unable to show statistically significant clinical benefit and further development was discontinued. The lack of clinical benefit has been attributed to several factors including the insufficient cytotoxic potency of doxorubicin, the instability of the hydrazone linker and the expression of Lewis Y by several normal tissues. (7–9).

CMD-193, which was developed by Wyeth Pharmaceuticals, Inc, was a humanized antibody (hu3S193) against the Lewis Y antigen that was conjugated to the DNA synthesis inhibitor, N-acetyl gamma calicheamicin dimethyl hydrazide (Calicheamicin) using the acid labile 4-(4′-acetylphenoxy) butanoic acid) linker (10). In preclinical studies, CMD-193, like SGN-15, was able to kill Lewis Y expressing tumors in both *in vitro* cytotoxicity studies and *in vivo* tumor efficacy studies (10) . In a phase I clinical study, myelosuppression and prolonged liver uptake which affected liver function were the most significant adverse events (11). Further clinical development of CMD-193 was terminated.

Gemtuzumab Ozogamacin (Mylotarg), which was also developed by Wyeth Pharmaceuticals, Inc (now Pfizer) and Celltech (now a part of UCB Brussels), was a humanized anti-CD33 IgG4 antibody, conjugated to Calicheamicin *via* the acid labile 4-4′-acetylphenoxy butanoic acid linker. Mylotarg had an average of 3 molecules of Calicheamicin per antibody and approximately 50% of the antibody did not contain Calicheamicin (12). Despite the high amount of unconjugated antibody, which could reduce its effectiveness, Mylotarg was able to kill CD33 expressing cells in *in-vitro* cytotoxicity studies and in preclinical *in-vivo* tumor efficacy studies (13). Mylotarg showed promise in clinical studies and was the first ADC to gain approval from the food and drug administration (FDA) in May 2000. Despite the promising clinical data, Mylotarg was eventually withdrawn from the market, in 2010, due to a lack of efficacy and an increased number of deaths in acute myelogenous leukemia (AML) patients as compared to patients treated with the standard of care chemotherapeutics (14).

Subsequent preclinical efforts to develop ADCs focused on the identification of potent cytotoxic drugs, linker chemistries that provide sufficient *in vitro* and *in vivo* stability and refinement of conjugation conditions to reduce the amount of unconjugated antibody.

In 2011 Adcetris (Brentuximab vedotin) was approved by the FDA to treat systemic anaplastic large cell lymphoma (sALCL) and Hodgkin’s lymphoma (HL). Adcetris is a chimeric IgG1 antibody against CD30 that is conjugated to an average of four molecules of the cytotoxic drug, monomethyl auristatin E (MMAE), *via* the thiols on cysteines using a valine-citrulline dipeptide linker with a p-aminobenzyl alcohol (PABA) spacer. MMAE is a potent inhibitor of cell growth *in vitro*. MMAE’s average *in vitro* IC_50_ values, against a panel of human tumor cell lines, were commonly below 1 nM as opposed to doxorubicin which had an average IC_50_ value of 631 nM (15).

In 2013, Kadcyla (T-DM1) was approved by the FDA to treat Her2 expressing metastatic breast cancer patients. Kadcyla is a humanized IgG1 antibody against Her2 that is conjugated to an average of 3.5 molecules of the cytotoxic maytansine drug, N2′-deacetyl-N2′-(3-mercapto-1-oxopropyl)-maytansine (DM1), *via* the amines on lysines using the 4-[N-maleimidomethyl] cyclohexane-1-carboxylate (MCC) linker. DM1’s average *in vitro* cytotoxicity IC_50_ values against several tumor cell lines were less than 1 nM (16,17).

**Fig. 1:**
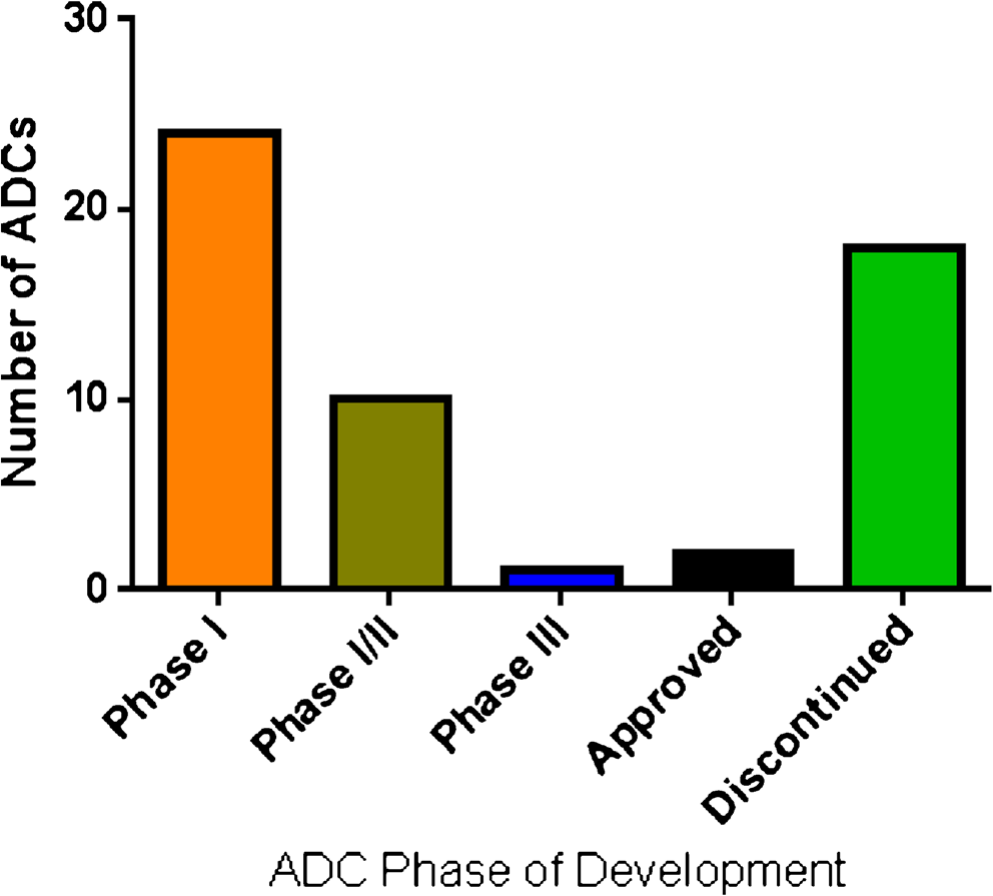
Clinical development of ADCs.

The clinical development of ADCs is expanding rapidly. Over 30 ADCs are in clinical development (Table I). The majority of ADCs are in either Phase I, Phase II or have active clinical efforts in both Phase I and Phase II (Fig. 1). Only one ADC, Inotuzumab Ozogamicin, has an active Phase III clinical effort. Over 15 ADCs have been withdrawn from clinical development for various reasons, which include a lack of anti-tumor response, unacceptable toxicity or a change in company strategy. The results from the clinical development of ADCs influence the preclinical development efforts, which lead to the creation of the next generation of ADCs.

**Table I:** ADCs in Clinical Development

ADC	Target	Phase of Development	Indication	Isotype	Payload
ABT-414	Activated EGFR/EGFRvIII	Phase I	GBM, SCC	IgG1	MMAF
AGS16M8F	ENPP3	Phase I	RCC	IgG2	MMAF
ASG22M6E	Nectin 4	Phase I	Solid tumors	IgG1	MMAE
AGS67E	CD37	Phase I	NHL, CLL, AML	IgG2	MMAE
AMG 172	CD27L	Phase I	RCC	IgG1	DM1
AMG-595	EGFRvIII	Phase I	GBM	ND	DM1
ASG15E	SLITRK6	Phase I	Solid tumors	IgG2	MMAE
Bay 94-9343	Mesothelin	Phase I	Solid tumors	IgG1	DM4
BT-062	CD138	Phase I/II	Multiple myeloma	IgG4	DM4
Glembatumumab Vedotin (CDX-011)	GPNMB	Phase II	Breast cancer	IgG2	MMAE
IMGN-853	FOLR1	Phase I	Solid tumors	IgG1	DM4
IMMU-130	CEACAM5	Phase I	Colorectal	IgG1	SN-38
IMMU-132	TACSTD2 (TROP2)	Phase I	Epithelial cancer	IgG1	SN-38
IMGN529	CD37	Phase I	NHL, CLL	IgG1	DM1
Inotuzumab Ozogamicin (CMC-544)	CD22	Phase III	ALL	IgG4	CalichDMH
MDX-1203	CD70 (MDX-1115)	Phase I	Clear cell RCC, B-NHL	ND	CC-1065
Milatuzumab	CD74	Phase I/II	Multiple myeloma	IgG1	Doxorubicin
MLN0264	Guanylyl cyclase C	Phase I	Colorectal	ND	MMAE
PF-0626350	5T4	Phase I	Solid tumor	ND	MMAF
PSMA (BrUOG 263)	PSMA	Phase I/II	Prostate	IgG1	MMAE
RG7450 (DSTP3086S)	STEAP1	Phase I	Prostate	ND	MMAE
RG7458 (DMUC5754A)	MUC16	Phase I	Ovarian	IgG1	MMAE
RG7593 (DCDT2980S)	CD22	Phase I	NHL	IgG1	MMAE
RG7596 (DCDS4501A)	CD79b	Phase I/II	NHL	IgG1	MMAE
RG7598	ND	Phase I	ND	ND	Undisclosed
RG7599 (DNIB0600A)	NaPi2b	Phase I	Ovarian, NSCLC	IgG1	MMAE
RG7600	ND	Phase I	ND	ND	ND
RG7636	Endothelin B receptor	Phase I	Melanoma	ND	MMAE
SAR3419	CD19	Phase I	DLBCL, ALL	IgG1	DM4
SAR566658	CA6	Phase I	Solid tumor	IgG1	DM4
SC16LD6.5	Fyn3	Phase I/II	SCLC	ND	D6.5
SGN-CD19A	CD19	Phase I	ALL, NHL	ND	MMAF
SGN-CD33A	CD33	Phase I	AML	IgG1	PBD (SGD-1882)
SGN-LIV1A	LIV-1	Phase I	Breast cancer	ND	MMAE

*ND* not disclosed, *SN-30* 7-ethyl-10-hydroxycamptothecin, *PBD* Pyrrolobenzodiazepine, *MMAE* Monomethylauristatin E, *MMAF* Monomethylauristatin F, *GBM* Glioblastoma multiforme, *RCC* Renal cell cancer, *SCLC* Small cell lung cancer, *NSCLC* Non-small cell lung cancer, *AML* Acute Lymphoblastic Leukemia, *NHL* Non-hodgkin lymphoma, *ALL* Acute lymphoblastic leukemia, *DLBCL* Diffuse large B-cell lymphoma, *SCC* Squamous cell carcinoma

**Fig. 2:**
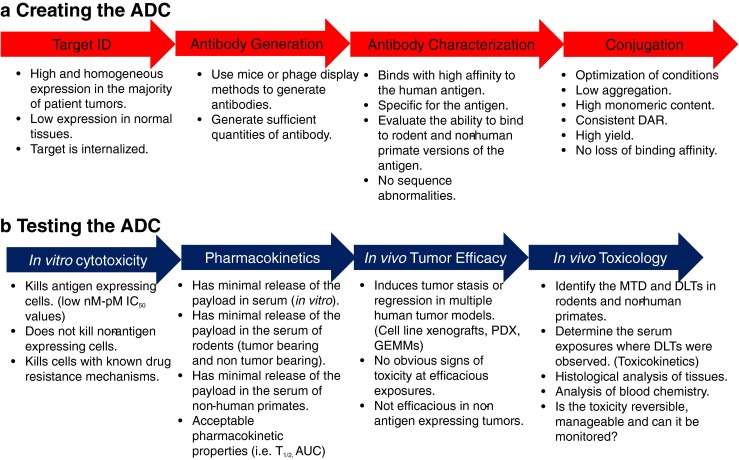
Preclinical development and evaluation of ADCs.

There are several key areas of focus for the preclinical development of ADCs. These areas include the selection of an ADC target, the selection of an appropriate antibody and the selection of appropriate cytotoxic drugs and linkers. Furthermore, attention has to be given to characterizing the physiochemical properties of the ADCs, evaluation of the *in vitro* cytotoxicity, the *in vivo* anti- tumor efficacy of the ADCs, the pharmacokinetic properties of the ADCs and their toxicology/safety profiles (Fig. 2).

## Target Selection

The optimal selection criteria for cell surface tumor antigens are that the antigen should be expressed selectively and at higher levels by tumors than normal tissues and the antigens are internalized by the tumor cell. Furthermore it would be preferred if the tumor antigens are expressed homogeneously to increase the number of cells killed by the ADC. It is unclear what the threshold receptor density or the internalization rate should be for a tumor antigen because the receptor density and the internalization rates may vary depending on the tumor antigen and tumor type. Thus far three ADCs have been approved to treat cancer patients. Those ADCs are Gemtuzumab Ozogamicin (Mylotarg), which targets CD33 expressing tumors; Adcetris, which targets CD30 expressing tumors and Kadcyla, which targets Her2 expressing tumors (Table II).

**Table II:** List of Approved ADCs

ADC	Target	Isotype	Payload	Indication	Date of Approval
Mylotarg	CD33	IgG4	Calicheamicin	AML	2000 (US) ^a^
Kadcyla (T-DM1)	Her2	IgG1	DM1	Her2 positive late stage breast	2013 (US)
Adcetris (SGN-35)	CD30	IgG1	MMAE	sALCL, relapsed/refractory Hodgkin lymphoma	2011 (US)

*AML* Acute Lymphoblastic Leukemia, *ACLC* Anaplastic large cell lymphoma

^a^ Withdrawn in 2010

### CD33

CD33 (Siglec-3) is expressed by the precursor myeloid and monocytic cells. CD33 is expressed in approximately 85–90% of adult and child AML patients and in 100% of acute promyelocytic leukemia (APL), which is a subset of AML (18,19). CD33 expression correlates with poor prognosis for children with ALL (20). High levels of CD33 expression have been reported in the bone marrow of AML patients while substantially lower levels of expression have been observed in normal bone marrow (21). The restricted expression of CD33 in normal tissues and the high levels of expression in AML made CD33 an attractive therapeutic target.

Three ADCs, Mylotarg, AVE9633, and SGN-CD33A, have been developed to treat patients with CD33 expressing tumors.

AVE9633 was a humanized IgG1 antibody conjugated to an average of 3–5 molecules of N2′-deacetyl-N2′-(4-mercapto-4-methyl-1-oxopentyl)-6-methylmaytansine (DM4) *via* a hindered disulfide linker on lysines. In preclinical studies, AVE9633 selectively killed AML cell lines and AML patient samples *in vitro* (22,23). Three phase I studies were performed and a total of 54 AML patients were treated with AVE9633. Patients were treated on day 1 of a 21 day cycle (Day 1), days 1 and 8 (Day 1/8) and days 1, 4 and 7 (Day1/4/7) of a 28 day cycle. The day 1 and day1/4/7 schedules were terminated early due lack of cytotoxic activity at doses higher than the doses that saturated binding to CD33 (22).

Mylotarg, as was previously mentioned, was removed from the market in 2010, which leaves SGN-CD33A as the only ADC currently in clinical development against CD33. SGN-CD33A is composed of humanized IgG1 antibody (h2H12), which contains two engineered cysteines at position 239 (S239C) on the antibody heavy chains. The DNA synthesis inhibitor, made by the former Spirogen LTD (now Astra-Zeneca/MedImmune), pyrrolobenzodiazepine (PBD), is covalently conjugated to each site specific cysteine using the maleimidocaproyl-valine-alanine linker. SGN-CD33A is the first publically disclosed site specific ADC in clinical development.

### CD30

CD30 (TNFRSF8) is expressed on activated T and B cells and a small population of eosinophils. It is also expressed in sALCL, HL, mature T cell lymphomas and B cell derived non-Hodgkins lymphoma (NHL). sALCL is a rare type of NHL, comprising approximately 3% of all NHL but is one of the more common T cell lymphomas (24). CD30 is expressed uniformly on sALCL (25). A soluble form of CD30 has been reported in the sera of cancer patients but this does not seem to have a deleterious effect on Adcetris (26). Limited expression of CD30 has been reported in normal tissues and high expression has been reported in sALCL, HL and other malignancies (27).

Adcetris had objective response rates of 75% for Hodgkin’s lymphoma (HL) patients and 86% for sALCL patients. Tumor reductions were reported in 94% of the HL patients and 97% of the sALCL patients (28). Interestingly CD30 expression in diffuse large B cell lymphoma (DLBCL) patients did not correlate with patient response to Adcetris. Tumor responses were observed in DLBCL patients that had undetectable expression of CD30 *via* immunohistochemical evaluation (29). These data suggest that CD30 expression alone in DLBCL patients may not predict response to Adcetris. It also suggests that low CD30 expression is sufficient for responses to Adcetris.

### Her2 (ERBB2)

Unlike CD33 and CD30, which are predominately expressed by hematological malignancies, Her2 is predominantly expressed by solid tumors. In particular, it is expressed in breast, gastric and ovarian cancer amongst others. Her2 is over expressed by approximately 25% of patients with invasive breast cancer. It is uniformly expressed in breast cancer and high expression of Her2 correlates with poor prognosis (30–33). Her 2 is also expressed by several normal tissues including, the skin, respiratory tract, heart, gastrointestinal epithelial cells and in the urinary and reproductive tracts (34). Kadcyla, also known as T-DM1, was approved in 2013 for Her2 expressing late stage breast cancer patients. The objective response rate in the pivotal EMILIA trial was 43.6% for patients treated with Kadcyla and 30.8% for patients treated with lapatinib and capecitabine (35).

### Solid Tumor Antigens

Developing antibody therapeutics for targets expressed in solid tumors may offer some additional challenges that may not exist for hematological cancer targets. For hematological cancers, it is possible to determine the dose that achieves receptor saturation. Once that dose is achieved, the addition of higher amounts of ADC would not be beneficial. Antibody delivery to solid tumors, however, is very complex. Solid tumors have high intratumoral pressures, due to the lack of lymphatics (36). Solid tumors have leaky blood vessels and chaotic blood flow, which can result in heterogeneous distribution of the antibody within the tumor thus rendering portions of the tumor inaccessible to the antibody or ADC (36–38). Another factor to consider is the large size of antibodies, which have a molecular weight of ~150 kD. The large size of the antibodies may limit their ability to efficiently penetrate deep within a solid tumor mass thus leaving portions of the tumor untreated and free to continue to grow (39). In addition to the physical properties of the antibody, the expression of the antigen must also be taken into consideration. If the tumor antigen is heterogeneously expressed within the tumor, portions of the tumor could be devoid of ADC thus allowing portions of the tumor to be untreated, which enables tumor growth. Lastly antibody affinities may impact anti-tumor efficacy of an ADC. Antibodies with high affinities are tightly bound to the antigen and may be unable to penetrate the tumor as well as antibodies with lower affinities, which will reduce the effectiveness of the ADC (40,41).

The current operating hypothesis is an ADC must be internalized in order for the drug to be released from the antibody and subsequently kill the antigen expressing cell. This has resulted in linkers that are designed to release their drug in a low pH environment or upon proteolysis using lysosomal enzymes. Although this is one way ADCs function, it is possible that some ADC drugs could be released without being internalized. The anti-CEACAM5 ADC (Epratuzumab-SN-38), which uses the CL2A linker combined with the SN-38 drug, has been reported to deliver the ADC to the antigen expressing tumors *in vivo* but releases the SN-38 drug without being efficiently internalized (42). The authors state that SN-38 is slowly released from the anti-CEACAM5 antibody (@50%/day) and the *in vitro* internalization rate for the anti-CEACAM5 antibody is slow. However, it is not clear that *in vitro* rates of internalization necessarily predict *in vivo* rates. Thus, the use of pH sensitive dyes (*i.e.* pHRodo^TM^) or far red dyes to label ADCs to evaluate the internalization and intracellular trafficking of ADCs *in vivo*, could be a useful tool to determine the importance of internalization to *in vivo* efficacy.

## Selecting the Antibody

### Antibody Isotype

The first ADCs were developed using murine antibodies. Patients developed HAMA responses against the murine antibody, which may have limited the effectiveness of these ADCs (3). The majority of the ADCs currently in clinical development are chimeric, fully human or humanized IgG1 antibodies. IgG2 and IgG4 isotypes are also being developed as ADCs (Table I).

**Fig. 3:**
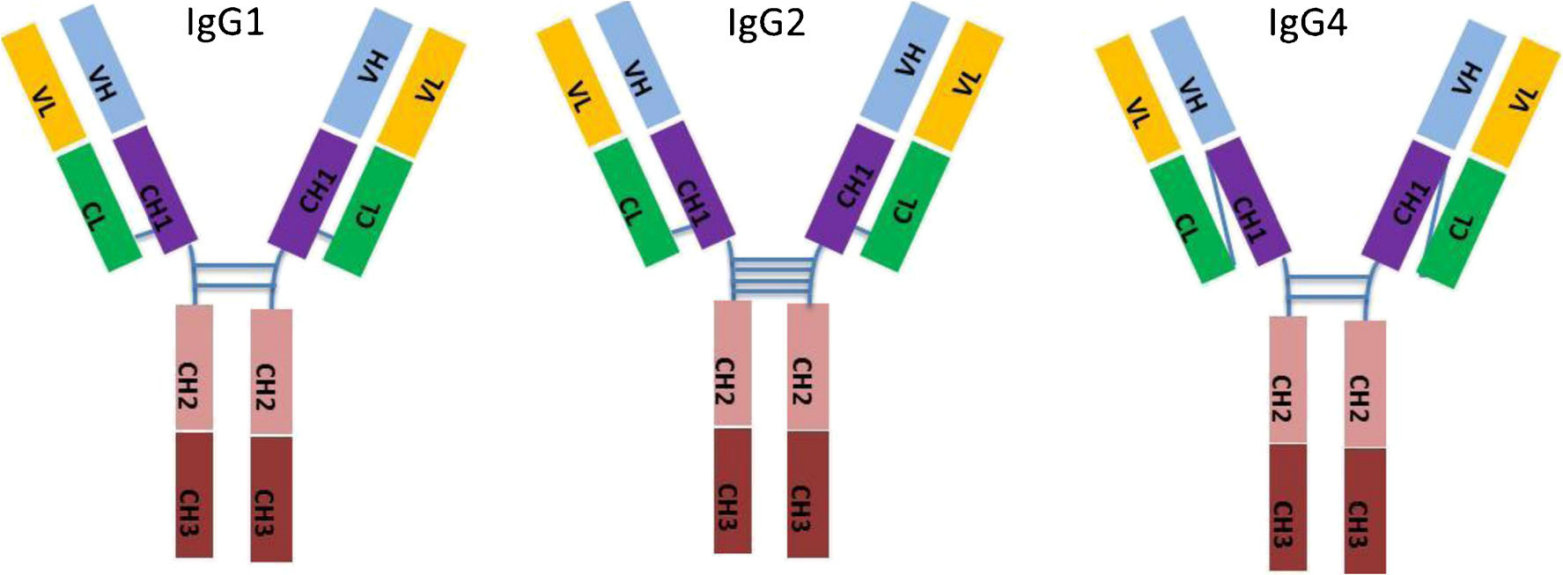
IgG isotypes used as ADCs.

There are structural differences between the various antibody isoforms (Fig. 3). The IgG2 isotype has four interchain disulfide bonds in the hinge region while the IgG1 and IgG4 isoforms have two interchain disulfide bonds (Fig. 3). The conjugation of the cytotoxic drug to the antibody, on cysteines, requires a partial reduction of the disulfides in the hinge region of the antibody. Tris (2-carboxyethyl)-phosphine (TCEP) is commonly used to reduce the antibody thus increasing the number of free thiols available for conjugation. IgG2 antibodies require higher concentrations of TCEP and longer reaction times than is required for IgG1 antibodies (43). Furthermore the IgG2 isoform exists as an A form, a B form and a hybrid A/B form (44). The significance of how these different IgG2 isoforms affect the production or conjugation of a drug to an ADC is unclear.

Some IgG1 antibodies have antibody-dependent cellular cytotoxicity (ADCC) and complement dependent cytotoxicity (CDC), which is achieved through binding of the antibody’s Fc domain to the Fcγ receptors on natural killer cells, monocytes and macrophages (45). IgG2 and IgG4 antibodies have little to no CDC activity (46). Although IgG1 antibodies, such as Trastuzumab, can have ADCC and CDC activities, it is unclear whether these activities are important for an ADC (47). To address the potential differences between IgG1, IgG2 and IgG4 ADCs, an anti-CD70 ADC, which was conjugated to valine-citrulline monomethyl auristatin F (MMAF) (43). The authors also mutated the Fc domains of the anti-CD70 IgG1 (IgG1v1) and IgG4 (IgG4v1) ADCs to reduce binding affinity to the Fcγ receptors. The IgG1v1-vc4 ADC had better *in vivo* tumor efficacy than the native IgG1-vcF4 ADC, which was attributed to increased serum exposure of the Fc mutated IgG1 ADC. This provided only a partial explanation for the enhanced efficacy of the Fc mutated IgG1 ADC because the wild type IgG2-vcF4 ADC had comparable serum exposure as the Fc mutated IgG1 ADC yet it was not as efficacious as the mutated IgG1 ADC.

## Cytotoxic Drugs and Linkers

### Cytotoxic Drugs

The drugs used for ADCs inhibit cellular processes that are vital for cell proliferation and/or survival, such as tubulin polymerization or DNA replication (Table III). Inhibition of tubulin function or DNA synthesis induces apoptotic cell death. The current cytotoxic drugs, used as ADC payloads, are typically 100–2,000 fold more potent than doxorubicin, vinca alkaloids or taxanes (48–51). Although the majority of drugs in clinical development inhibit tubulin function or DNA synthesis, new drugs which inhibit critical cellular processes, such as RNA synthesis or membrane disruption are being developed as ADC payloads and may enter into clinical development. The cytotoxic drugs or their metabolites have varying degrees of membrane permeability. MMAE, for example, is membrane permeable and when released from a cell it may kill neighboring cells by a process known as bystander effect (52). MMAF has reduced membrane permeability and the metabolite, Cys-mcMMAF, does not exhibit bystander activity. Bystander activity may be useful in a solid tumor setting when the target antigen is heterogeneous expressed in the tumor. An anti-mesothelin ADC, Anetumab ravtansine, which is a fully human anti-mesothelin antibody conjugated to an average of 3.2 molecules of the cytotoxic maytansine drug DM4, was reported to exhibit bystander activity in tumor xenograft models (53). Tumor regressions were observed in tumor models where only 20% of the cells expressed mesothelin, which suggests that the non-mesothelin cells were killed by the release of the DM4 metabolite from the mesothelin expressing cells.

**Table III:** ADC Payloads

Drug/Payload (Payload name)	Mechanism of action	Stage of development
Auristatin (MMAE/MMMAF)	Tubulin assembly inhibitor	Approved/Clinical
Maytansinoid (DM1/DM4)	Tubulin assembly inhibitor	Approved/Clinical
Tubulysin	Tubulin assembly Inhibitor	Preclinical
SN-38	Topoisomerase inhibitor	Clinical
Calicheamicin	DNA synthesis inhibitor	Clinical
Doxorubicin	DNA synthesis inhibitor	Clinical
Pyrrolobenzodiazepine (SGD-1882)	DNA synthesis inhibitor	Clinical
Duocarmycin (CC1065)	DNA synthesis inhibitor	Clinical
D6.5	DNA synthesis inhibitor	Clinical
α- Amanitin	RNA polymerase II inhibitor	Preclinical

### Linkers

Linkers are grouped into two classes (Table IV). They are either cleavable or non-cleavable. Cleavable linkers can be dipeptide linkers, such as valine-citrulline, and are cleaved by proteases (*i.e.* Cathepsin B), which results in the release of the cytotoxic drug. Cleavable linkers can also be acid labile, such as hydrazones and hindered disulfides, where the lysosomal environment results in the release of the cytotoxic drug. ADCs with non-cleavable linkers are degraded in the lysosome, which results in the formation of an amino acid-drug combination (*i.e.* Cys-mcMMAF). Although the linkers can be processed by the enzymes or environment of the lysosome, some linkers such as maleimide linkers, can transfer the cytotoxic drug to free sulfhydryls such as the cysteine on human serum albumin (HSA) or glutathione *via* a retro-michael reaction (54,55). This can cause a 25% or greater decrease in the tumor exposure of the ADC (54). This decrease in tumor exposure may reduce the effectiveness of the ADC.

**Table IV:** Linkers Used for ADCs

Linker name	Cleavable/Non-cleavable	Conditions for payload release	Payload
Valine-citrulline (vc)	Cleavable	Protease	MMAE
SPP	Cleavable	Reducing/Oxidizing environment	DM1
SPDB	Cleavable	Reducing/Oxidizing environment	DM4
6-maleimidocaproyl hydrazide	Cleavable	Acid labile	Doxorubicin
4-(4′-acetylphenoxy) butanoic acid)	Cleavable	Acid labile	Calicheamicin
CL2A	Cleavable	pH mediated	SN-38
SMCC	Non-cleavable	Antibody degradation	DM1
Maleimidocaproyl (mc)	Non-cleavable	Antibody degradation	MMAF

*SPP* N-succinimidyl 4-(2-pyridyldithio)pentanoate, *SMCC* N-succinimidyl 4-(N-maleimidomethyl)cyclohexane-1 carboxylate, *SPDB* N-succinimidyl-4-(2-pyridyldithio)butyrate, *CL2A* maleimido-[short PEG]-Lys-PABOCO-20-O-SN-38

The linker/drug combination can influence the pharmacokinetics and the toxicology profile of the ADC. A humanized antibody (huC242) against CanAg/MUC1 was conjugated to the SPP-DM1 and SPDB-DM4 linker/drug combinations and evaluated in preclinical and clinical studies (Table V). The serum half-life in humans for the huC242-SPP-DM1 (Cantuzumab Mertansine), using a Q3W dosing schedule, ranged between 18 and 48.5 hrs while the serum half-life for huC242-SPDB-DM4 (IMGN242) was between 60 and 120 hrs (56). In addition to the apparent differences in the half-lives of the ADCs, the dose limiting toxicities (DLT) were also different. The DLT for the huC242-SPP-DM1 ADC was elevated hepatic transaminase and the DLT for the huC242-SPDB-DM4 ADC was ocular toxicity, which was only observed in patients with low serum levels of CanAg (56). These data suggest that the different drug linker combinations can have significant effects on the pharmacokinetics of the ADC and the toxicology profiles. Interestingly, several ADCs have been reported to have reversible ocular toxicity in patients (Table VI). Ocular toxicity has been observed primarily for ADCs conjugated to DM4 or MMAF but Kadcyla, which is conjugated to DM1, reported ocular toxicity in 31.3% of the patients in a phase II study (57). These observations have resulted in the inclusion of ophthalmologic evaluation in preclinical toxicology studies. A patent application, US 20120282282 A1, was filed by Immunogen where the claims suggest that the ocular toxicity for DM4 ADCs can be decreased by modifying the charge of the linker. The uncharged SPDB linker was reported to display decreased ocular toxicity in a rabbit model than the charged sulfo-SPDB linker.

**Table V:** Clinical Summary of huC242 ADCs

Antibody	Payload	Linker	Schedule	MTD (mg/m^2^)	T_1/2_ (hours)	DLT	Clinical Phase
huC242	DM1	SPP	Q1W	115	13–41	Elevated hepatic transaminase	I
huC242	DM1	SPP	Q3W	235	18–48.5	Elevated hepatic transaminase	I
huC242	DM4	SPDB	Q3W	168	60–120	Reversible ocular toxicity	I
huC242	DM4	SPDB	Q3W	168	N/A	Reversible ocular toxicity	II

**Table VI:** ADCs with Ocular Toxicity in the Clinic

Antibody	Target	Linker	Payload
SAR3419	CD19	SPDB	DM4
IMGN853	Folate Receptor a	SPDB	DM4
SGN-75	CD70	mc	MMAF
SAR566658	CA6	SPDB	DM4
BT-062	CD138	SPDB	DM4
huC242-DM4	CanAg	SPDB	DM4
SGN-CD19A	CD19	mc	MMAF
Kadcyla	Her2	SMCC	DM1

### Drug Resistance

One of the reasons why cancer therapies fail is due to drug resistance (58). There are several mechanisms responsible for resistance to chemotherapies, which includes the expression of drug-efflux pumps (59). Drug-efflux pumps lower the intracellular concentration of the drug which renders the drug ineffective. The expression of several drug efflux pumps including multi-drug resistance 1(MDR1/ABCB1), multi-drug resistance protein 1(MRP1/ABCC1) and breast cancer resistance protein (BCRP/ABCG2) have been implicated in resistance to various chemotherapeutics. Several cytotoxic drugs, used for ADCs, such as MMAE, DM1 and Calicheamicin, are known substrates of the drug efflux pump MDR1 (ABCB1) (60,61). MDR1 substrates tend to be hydrophobic drugs therefore modifying the metabolites to be polar or negatively charged may reduce the drugs from being pumped out of the cells *via* MDR1 (62).To counteract the MDR1 drug efflux pump, the linker used to conjugate DM1 to an anti-EpCam antibody was changed from N-succinimidyl-4-(maleimidomethyl) cyclohexane-1-carboxylate (SMCC) to the hydrophobic linker PEG_4_Mal. The resulting metabolite, lysine-PEG4Mal-DM1 was able to kill MDR1 expressing cells while the lysine-SMCC-DM1 was unable to kill MDR1 expressing cells (63). These data suggest that selection of the appropriate linker may be important to avoid resistance mediated *via* drug efflux pumps. In addition to selecting the appropriate linker to avoid drug efflux pumps, cytotoxic drugs that are not substrates for efflux pumps, such as PBDs, may also be considered. SGN-CD33A is conjugated to the PBD,SGD-1882, which is not an MDR1 substrate. SGN-CD33A can kill MDR1 expressing CD33 positive AML patient samples and is currently in Phase I.

Several methods, using fluorescent substrates for the various drug efflux pumps, have been used to assess the activity of the pumps and may be useful in characterizing the contributions of the various pumps to resistance to the ADC metabolites (64). Rhodamine 123, for example, can be used to assess drug efflux pump activity in cell lines and specific inhibitors of various efflux pumps can be used to determine the specific drug efflux pump’s contribution to the efflux activity in a cell line or patient derived cells (65).

## Antibody Conjugation Options

There are two methods used to covalently attach a cytotoxic drug to an antibody. Currently, the more conventional method, conjugates the cytotoxic drugs to an antibody’s solvent exposed cysteine or lysine residues. The site specific conjugation method conjugates the cytotoxic drugs to natural or non-natural amino acids that have been engineered at different locations on the antibody or to glycosyl moieties.

### Conventional Conjugation

The majority of the ADCs in clinical development are in this category. The cytotoxic drugs are conjugated to antibodies *via* the thiols on solvent exposed cysteines or the amines on lysines. The conjugation of cytotoxic drugs to the antibodies results in a heterogeneous mixture of ADCs where the number of cytotoxic drugs conjugated to an antibody typically varies from 0 to 8 drugs per antibody. Antibodies with a DAR of 8 or more have a higher clearance rate than antibodies with a lower DAR (11). The majority of the ADCs have an average DAR of approximately 4 drugs per antibody, which offers an acceptable balance between pharmacokinetic properties, toxicities and anti-tumor efficacy.

### Site Specific Conjugation

Cytotoxic drugs are conjugated to amino acids, typically cysteines or non-natural amino acids, which have been incorporated at distinct locations on the antibody. The resulting ADC is homogeneous composition where all of the antibodies contain a defined number of drugs per antibody. Typically a DAR of 2 is used although higher DARs could be achieved. The incorporation of a cytotoxic drug on specific positions of the antibody can influence the pharmacokinetic properties of the ADC. Several examples of site specifically conjugated ADCs, using antibodies containing engineered cysteines or the non-natural amino acid, p-acetyl phenyalanine (pAF), have reported improved *in vitro* and *in vivo* serum stability, improved pharmacokinetic properties, comparable anti-tumor efficacy and improved toxicology profiles as compared to the conventional ADCs (55,66,67). Cytotoxic drugs conjugated to pAF *via* an oxime bond are reported to have improved serum stability when compared to cytotoxic drugs conjugated to thiols *via* maleimide (10,55).

One cysteine engineered site specifically conjugated ADC, SGN-CD33A, is currently in phase I clinical development. This is probably the first of many site specific ADCs that will be evaluated in the clinic.

A couple of novel approaches for site-specific conjugation not requiring molecular engineering of the antibody are cysteine bridging approaches, which can specifically conjugate all of the cysteines with a defined DAR or ½ the number of cysteines (4 for a typical IgG1), and conjugation of glycosyl moieties.

There are several companies developing site specific conjugation technologies but most are currently in preclinical development (Table VII). This is an exciting area of development for ADCs.

**Table VII:** Companies Developing Site Specific ADC Technologies

Company	Type of Technology	Stage of Development
Ambrx Inc	Non-natural amino acid (NNAA)	Preclinical
Genentech/Roche, Novartis	Engineered cysteine (Thiomab)	Preclinical
Igenica Inc	(SNAP ADC) no mutation or NNAA	Preclinical
Meditope Bioscience Inc	Cetuximab derived residue grafts into Fab	Preclinical
Pfizer	FXIII substrate peptide - acceptor glutamine	Preclinical
Poly Therics Ltd	ThioBridge	Preclinical
Redwood Bioscience Inc (acquired by Catalent Pharma Solutions)	Aldehyde tag	Preclinical
Seattle Genetics	Engineered Cysteine	Clinical
Sutro Biopharma Inc	Non-natural amino acid	Preclinical
SynAffix	Glycosyl conjugation	Preclinical

## Challenges of Preclinical Development of ADCs

The data from clinical evaluation of ADCs has led to significant changes in the preclinical development of ADCs. The current cytotoxic drugs used as ADC payloads are more potent than methotrexate, doxorubicin and vinblastine, which were previously used as ADC payloads. The current sets of linkers, used to conjugate the drugs to the antibodies, are more stable than the previous linkers and the amount of unconjugated antibody has been dramatically reduced. These changes were incorporated into the preclinical development of the two FDA approved ADCs, Kadcyla and Adcetris.

The clinical development of more than 15 ADCs has been discontinued because the ADCs did not provide sufficient anti-tumor responses, had unacceptable toxicology profiles or the company decided to support other products in their pipeline. These ADCs apparently had an acceptable preclinical data package that supported the decision to progress them into clinical development. Inotuzumab Ozogamicin (CMC-544) has an active Phase III clinical effort and is currently the most advanced ADC in clinical development. CMC-544 showed significant inhibition of tumor growth in various preclinical human B-cell lymphoma cell line models (68). Pfizer, which is developing CMC-544, recently announced that it is discontinuing a Phase III trial in NHL but will continue the Phase III INO-VATE ALL study (B1931022) in ALL. This suggests that preclinical *in vivo* anti-tumor efficacy studies may not predict patient responses to the ADCs and additional preclinical studies may be warranted to increase the correlation between the data from the preclinical studies and clinical responses.

An area of focus in the preclinical development of ADCs could be in improving the tumor models used to evaluate the ADCs. Preclinical studies have suggested that human tumor xenografts, derived from cell lines, do not accurately reflect the original patient tumors they were derived from and may not predict clinical anti-tumor activity (69,70). Patient derived tumor xenografts (PDX) are derived from freshly isolated patient tumors that are implanted, grown and propagated in immunodeficient mice. A large panel of patient derived colon tumors was reported to accurately represent the histology and the range of molecular heterogeneity observed in colon cancer patients (71). The authors conducted an evaluation of cetuximab in the colorectal cancer PDX tumors and confirmed the key role of KRAS mutation in cetuximab resistance. The increased use of PDX models may provide better predictive value than the use of human tumor cell lines. ADCs against 5T4 and tissue factor have reported preclinical *in vivo* anti-tumor activity in PDX models (72,73).

Perhaps attention to the pharmacokinetics and pharmacodynamics of the ADC may add value to the preclinical effort. An evaluation of a PK/PD modeling approach reported that tumor growth rate and the ratio between the exposures and concentrations that induced tumor stasis were important in predicting tumor responses to Kadcyla (74). This PK/PD model was modified using the clinical PK data for Kadcyla and was used to predict the clinically efficacious exposures for an ADC against the 5T4 tumor antigen (75). The utility of this modeling approach is currently being evaluated.

The use of site specific ADCs, improvements in the pharmacokinetic properties of the ADCs through the development of better linkers and the use of potent cytotoxic drugs as ADC payloads combined with improvements in tumor model selection and target identification may result in higher success rates for ADCs in the clinic.

## Abbreviations

ALCL: Anaplastic large cell lymphoma
ADC: Antibody drug conjugate
ALL: Acute lymphoblastic leukemia
AML: Acute myeloid leukemia
CL2A: maleimido-[short PEG]-Lys-PABOCO-20-O-SN-38
DAR: Drug to antibody ratio
DLBCL: Diffuse large B-cell lymphoma
GBM: Glioblastoma multiforme
kD: Kilodalton
MMAE: Monomethylauristatin E
MMAF: Monomethylauristatin F
ND: Not disclosed
NHL: Non-hodgkin lymphoma
NNAA: Non-natural amino acid
NSCLC: Non-small cell lung cancer
PBD: Pyrrolobenzodiazepine
PDX: Patient derived xenograft
PK/PD: Pharmacokinetic/Pharmacodynamic
Q3W: Every 3 weeks
RCC: Renal cell cancer
SCC: Squamous cell carcinoma
SCLC: Small cell lung cancer
SMCC: N-succinimidyl 4-(N-maleimidomethyl)cyclohexane-1 carboxylate
SN-30: 7-ethyl-10-hydroxycamptothecin
SPDB: N-succinimidyl-4-(2-pyridyldithio)butyrate
SPP: N-succinimidyl 4-(2-pyridyldithio)pentanoate
